# 5-α reductase inhibitors and prostate cancer prevention: where do we turn now?

**DOI:** 10.1186/1741-7015-9-105

**Published:** 2011-09-15

**Authors:** Robert J Hamilton, Stephen J Freedland

**Affiliations:** 1 Division of urology, Department of Surgery, University of Toronto, 2075 Bayview Avenue, Toronto, ON M4N 3M5, Canada; 2 Duke Prostate Center, Division of Urologic Surgery, Departments of Surgery and Pathology, Duke University School of Medicine, Box 2626 DUMC, Durham, NC 27710, USA; 3 Section of Urology, Veterans Affairs Medical Center, Durham, NC, USA. 508 Fulton Street, Durham, NC 27710, USA

## Abstract

With the lifetime risk of being diagnosed with prostate cancer so great, an effective chemopreventive agent could have a profound impact on the lives of men. Despite decades of searching for such an agent, physicians still do not have an approved drug to offer their patients. In this article, we outline current strategies for preventing prostate cancer in general, with a focus on the 5-α-reductase inhibitors (5-ARIs) finasteride and dutasteride. We discuss the two landmark randomized, controlled trials of finasteride and dutasteride, highlighting the controversies stemming from the results, and address the issue of 5-ARI use, including reasons why providers may be hesitant to use these agents for chemoprevention. We further discuss the recent US Food and Drug Administration ruling against the proposed new indication for dutasteride and the change to the labeling of finasteride, both of which were intended to permit physicians to use the drugs for chemoprevention. Finally, we discuss future directions for 5-ARI research.

## Introduction

Prostate cancer is the most commonly diagnosed cancer among men and the second leading cause of cancer death [1]. With one in six men destined to be diagnosed with prostate cancer in their lifetimes and the costs associated with prostate cancer care being very high [2], the potential benefits of an effective chemoprevention agent are obvious [1]. Yet, despite decades of research in the field, there are still no approved pharmaceuticals for the prevention of prostate cancer. The 5-α reductase inhibitors (5-ARIs) finasteride and dutasteride are the most promising to date, but also the most controversial. Recently, the US Food and Drug Administration (FDA) ruled against proposals to add an indication to dutasteride and alter the labeling of finasteride that would allow prescribers to use these drugs for chemoprevention. The impact of this decision on the future of prostate chemoprevention remains to be seen. Is it the nail in the coffin or the needed wake-up call to turn the field in another direction?

In this article, we outline strategies for preventing prostate cancer in general, but focus specifically on the 5-ARIs. We discuss the two landmark randomized, controlled trials (RCTs) of finasteride and dutasteride and highlight the controversies stemming from the results. We address the issue of 5-ARI use and why providers may be hesitant to use these agents for chemoprevention, as well as the recent FDA ruling.

## Preventing prostate cancer

Over the years, several nutrients, lifestyle modifications and pharmaceutical agents have been studied as potential chemoprevention candidates [3]. Selenium and vitamin E showed promise [4,5]. However, these were definitively evaluated in the Selenium and Vitamin E Cancer Prevention Trial, and neither agent reduced prostate cancer risk [6]. Vitamin D analogs, nonsteroidal anti-inflammatory drugs (NSAIDs) and toremifene (a selective estrogen receptor modulator) have all been evaluated in laboratory and/or observational studies [7-9]. However, vitamin D has not been formally tested in primary prevention trials. An attempt was made to study the NSAID rofecoxib, but the trial was closed when the drug was taken off the market for safety reasons [10]. Toremifene showed a modest risk reduction in a phase II trial [11], but no significant risk reduction in a phase III trial [12].

Statin medications hold promise for prostate cancer prevention. They appear to reduce prostate-specific antigen (PSA) [13,14], and while they do not apparently reduce prostate cancer risk overall, they appear to preferentially reduce the risk of advanced or aggressive prostate cancer [15]. They are also associated with improved outcomes after radiation therapy [16] and radical prostatectomy [17], though data for the latter are conflicting [18]. The advantage of statins is their proven safety record and their welcome side effects of decreased cholesterol levels and cardiac disease risk reduction. Though no trial of the use of statins in primary prostate cancer prevention is currently underway, two studies of statins as secondary preventive agents are. One trial is randomizing patients to simvastatin or placebo prior to radical prostatectomy and is examining changes in benign and malignant tissue in the prostate specimen [19]. The second trial is a phase II study of atorvastatin and celecoxib in patients with rising PSA levels after definitive local therapy and is examining changes in biomarkers, including PSA [20].

Taken together, the medical community is unlikely to have a compound with proven ability to prevent prostate cancer emanate from these studies in the forseeable future.

## The 5-α reductase inhibitors

### Rationale and benefits

By far the most promising and well-studied chemopreventive agents are the 5-ARIs finasteride and dutasteride. The 5-α reductase (5-AR) enzyme is responsible for converting testosterone into dihydrotestosterone. Dihydrotestosterone is a prevalent and potent androgen in prostate tissue and is responsible for embryologic development of the prostate [21], growth of the prostate and promotion of prostate cancer [22]. Finasteride inhibits 5-AR type 2, and dutasteride inhibits 5-AR types 1 and 2. Both finasteride and dutasteride were designed and approved for the treatment of benign prostatic hyperplasia (BPH) and have proven efficacy in this regard [23-26].

Finasteride was studied in the Prostate Cancer Prevention Trial (PCPT) [27]. In this RCT of 18,000 men ≥ 55 years of age with a normal digital rectal examination (DRE) and PSA level ≤ 3 ng/mL, after seven years, those in the finasteride arm had a 25% reduction in prostate cancer incidence (18.4% vs. 24.4%; *P *< 0.001). Dutasteride was studied in the REduction by DUtasteride of Prostate Cancer Events (REDUCE) trial [28]. In this RCT of 6,729 men ages 50 to 75 years with a prior negative prostate biopsy who had at least one on-study biopsy, those in the dutasteride arm had a 23% reduction in prostate cancer incidence after four years (19.9% vs. 25.1%; *P *< 0.001). The two trials are compared in Table 1. These risk reductions translate into a number needed to treat to prevent one case of prostate cancer of 17 for finasteride and 20 for dutasteride. If the story ended there, many men would undoubtedly be taking a 5-ARI drug today.

**Table 1:** Comparison of the two randomized controlled trials of 5-α reductase inhibitors for primary prevention of prostate cancer^a^

	PCPT	REDUCE
Agent studied	Finasteride 5 mg	Dutasteride 0.5 mg
Manufacturer	Merck & Co., Inc.	GlaxoSmithKline
Enzyme inhibition	5-AR type 2	5-AR types 1 and 2
Study size	18,882	8,231
Final analysis size, (drug:placebo)	9,060 (4,368:4,692)	6,729 (3,305:3,424)
Follow-up	7 years	4 years
Eligibility criteria	Age ≥55 years	Age 50 to 75
	Normal DRE	PSA 2.5 to 10 ng/mL
	PSA ≤3 ng/mL	Prior negative prostate biopsy (6-core minimum) within 6 months
	AUA Symptom Score <20	AUA Symptom Score <25 (or <20 if taking α blockers)
		Excluded if
		HGPIN
		ASAP
		> 1 biopsy prior
		Gland volume > 80 cm^3^
In-study measures	Annual PSA, DRE	Semiannual PSA, DRE
	Finasteride PSA adjusted by 2× to 2.3×	Dutasteride PSA adjusted by 2×
	Triggers for biopsy	Triggers for biopsy
	Abnormal DRE	Not specified
	PSA > 4 ng/mL	Protocol biopsies at 2 and 4 years
	End-of-study biopsy offered to all without cancer after 7 years	
Biopsies for cause, %	39.4%	12.0%
Primary end point	Prostate cancer detection	Prostate cancer detection
	Finasteride 803 (18.4%)	Dutasteride 659 (19.9%)
	Placebo 1,147 (24.4%)	Placebo 858 (25.1%)
	RRR = 24.8%, 95% CI 18.6 to 30.6; *P *< 0.001	RRR = 22.8%, 95% CI 15.2 to 29.8, *P *< 0.001
Secondary end points	Prostate volume at biopsy	Change in prostate volume from years 1 to 4
	Finasteride = 25.5 cm^3^	Dutasteride 45.7 to 39.0 cm^3 ^= -17.5%
	Placebo = 33.6 cm^3^	Placebo 45.8 to 56.2 cm^3 ^= +19.7%
	Relative difference = 24.1%	Relative difference in final volume = 30.1%
		HGPIN
		Dutasteride 3.7%
		Placebo 6.0%
		RRR = 39.2%, 95% CI 24.2-51.1, p<0.001
		ASAP
		Dutasteride 3.8%
		Placebo 4.9%
		RRR = 21.2%, 95% CI 1.3-37.1, p = 0.04
High-grade disease	Gleason ≥7 detection	Gleason ≥7 detection
	Finasteride 280 (6.4%)	Dutasteride 220 (6.7%)
	Placebo 237 (5.1%)	Placebo 233 (6.8%)
	RR = 1.67, 95% CI 1.44-1.93, p = 0.005	RR = 1.02, p = 0.81
	Gleason ≥8 detection	Gleason ≥8 detection
	Finasteride 90 (2.1%)	Dutasteride 29 (0.9%)
	Placebo 53 (1.1%)	Placebo 19 (0.6%)
	RR = 1.90; 95% CI and *P *value not given	RR = 1.5, 95% CI not given, *P *= 0.15
NNT to prevent 1 cancer	17	20

^a^5-AR: 5-α reductase enzyme; 5-ARIs: 5-α reductase inhibitors; ASAP: atypical small acinar proliferation; AUA: American Urological Association; DRE: digital rectal examination; HGPIN: high-grade prostatic intraepithelial neoplasia; NNT: number needed to treat; RR: relative risk; RRR: relative risk reduction; 95% CI: 95% confidence interval.

### The controversy

Unfortunately, the results of the two primary prevention trials are more complicated. First, these trials have been criticized for lack of generalizability because the results were driven largely by end-of-study biopsies as opposed to biopsies clinically triggered by elevated PSA or DRE abnormalities. In fact, subgroup analyses of only biopsies triggered by clinical events suggested 5-ARIs achieved less impressive relative risk reductions (RRRs) (PCPT: 9% RRR; REDUCE: 1% RRR) [27,28]. It is argued that these risk reduction estimates more closely mirror what would be seen in general practice.

Second, it appears that 5-ARIs preferentially prevent low-grade cancers. In both trials, the overall cancer risk reduction was driven entirely by the reduction in Gleason ≤ 6 tumors. Such low-grade cancers are unlikely to lead to prostate cancer mortality and thus arguably do not warrant preventive efforts [29,30]. Indeed, a pathological review of cancers in the PCPT demonstrated that 40% of the Gleason ≤ 6 tumors met established pathologic criteria for clinically insignificant disease [31,32]. A similar analysis of the REDUCE trial cancers has yet been published; however, a pathologic review by an expert genitourinary pathologist commissioned by the FDA concluded that 80% of the Gleason ≤ 6 tumors met the criteria for clinically insignificant disease [33]. Two counterarguments are apparent: (1) In general, as many as 30% of cancers initially deemed insignificant on the basis of the first biopsy are reclassified as significant on the basis of a subsequent biopsy [34]; and (2) currently in the United States, > 90% of men diagnosed with Gleason 6 tumors undergo surgery or radiotherapy [35]. If these trends continue, reducing the incidence of these often-treated cancers with 5-ARIs may be meaningful.

By far the issue receiving the greatest concern from these two prevention trials is the increased risk of high-grade disease. In the PCPT, the proportion of high-grade tumors (Gleason ≥ 7) was 27% higher in the finasteride arm (280 (6.4%) vs. 237 (5.1%); *P *= 0.005), and in the REDUCE trial, though no significant difference in Gleason ≥ 7 tumors was reported (220 (6.7%) vs. 233 (6.8%); *P *= 0.81), there was clearly a trend toward increased risk in Gleason ≥ 8 tumors (29 (0.9%) vs. 19 (0.6%); *P *= 0.15), particularly in years 3 and 4 (12 (0.5%) vs. 1 (< 0.1%); *P *= 0.003).

Two theories have been suggested as to why higher-grade disease is noted in the 5-ARI arms: (1) 5-ARIs shrink prostate volume, thus making it more likely to find high-grade disease when it is present [36]; and (2) 5-ARIs, by reducing confounding from BPH, heighten the sensitivity of the PSA and DRE in the detection of high-grade disease [37,38]. In a *post hoc *analysis using imputation based on these two theories, the PCPT group concluded that there was actually a 27% reduction in the risk of Gleason ≥ 7 tumors, though there remained, albeit reduced, a 25% increase in risk of Gleason ≥ 8 tumors [39]. In the REDUCE study, the authors explained that the increased risk of Gleason ≥ 8 tumors was a product of more cancers being detected in the placebo group in years 1 and 2. That is, if these tumors were not detected and therefore not removed from analysis in years 3 and 4, a portion of them would have progressed to high-grade disease and would have balanced the higher-grade tumors seen in the dutasteride arm in years 3 and 4. However, the natural history of low-grade tumors dedifferentiating to high-grade tumors is not known and unlikely to be sufficiently rapid to explain the difference in years 3 and 4.

The fact remains that both trials observed at least concerning trends toward increased high-grade disease, though it should be noted that the absolute risk increase is small. The true extent to which these trends can be explained by the theories proposed is unknown, but for now concern lingers that 5-ARIs may induce or selectively promote growth of high-grade disease.

## Use of 5-α reductase inhibitors in practice

**Figure 1:**
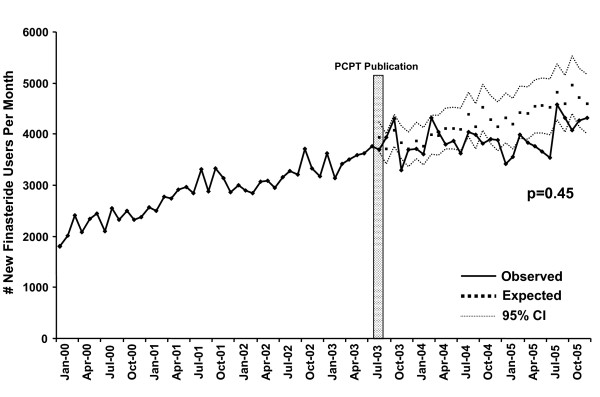
**Number of new finasteride users among Veterans Health Administration (VHA) patients from January 2000 to October 2005, before and after publication of the PCPT, adjusted for changes in the size and age of the male VHA population over time (adapted from Hamilton *et al***. [40]).

The only study exploring the use of 5-ARIs in clinical practice observed that while use slowly increased from 2000 to 2005 in the Veterans Health Administration, there was a subtle trend toward decreased use after publication of the PCPT (Figure 1) [40]. This change did not reach statistical significance, and prescriptions could not be classified by intended use (prostate cancer prevention vs. treatment of BPH).

In the accompanying survey, urologists cited concerns over the risk of high-grade disease as the most common reason not to prescribe 5-ARIs for chemoprevention [40]. This article was written prior to the publication of the REDUCE trial. As such, the extent to which 5-ARIs are currently being used for prostate cancer prevention remains unclear.

Most recently, the FDA Oncology Drugs Advisory Committee (ODAC) reviewed applications by GlaxoSmithKline to add an indication for dutasteride for the prevention of prostate cancer in men at increased risk for prostate cancer and by Merck to alter the labeling for finasteride to reflect a more favorable safety profile with regard to preventing prostate cancer. The FDA conducted its own reanalysis of the PCPT and REDUCE trial results and concluded that (1) the risks of high-grade cancer were likely real and could not be explained entirely by volume grade bias, increased sensitivity of PSA and DRE or removal of low-grade cancers in the REDUCE trial placebo arm, (2) the majority of cancers prevented were low risk and the trials provided no evidence of 5-ARI treatment and prostate cancer mortality reduction and (3) the results were not generalizable to the US population because end-of-study biopsies do not mirror clinical practice. The FDA ODAC voted against the new indication for dutasteride (yes = 2, no = 14, abstain = 2) and against the new labeling for finasteride (yes = 0, no = 17, abstain = 1). GlaxoSmithKline has subsequently announced that it is withdrawing applications for similar approval in other countries [41].

Though we do not have an up-to-date assessment of whether 5-ARIs are being prescribed for chemoprevention, it is likely, given trepidation before, that the FDA ruling will lead to more hesitation in prescribing 5-ARIs for this indication. Apparent from the FDA ODAC meeting was that even among people who study the issue of 5-ARI chemoprevention extensively, eight years after publication of the PCPT there is still vast disagreement regarding the benefits and harms. With such disagreement among experts, it is not surprising that practicing physicians do not have a clear answer when their patients ask about 5-ARIs.

## Cost-effectiveness

Since the PCPT, 11 publications have explored various aspects of the trade-offs among the benefits, harms and costs of 5-ARI chemoprevention for prostate cancer. An extensive analysis of these studies is beyond the scope of this review and is complicated by the fact that each is based on a set of assumptions about the PCPT data. For example, some model the risk of high-grade disease at face value from the trial; others impute less risk of high-grade disease based on the volume grade and PSA sensitivity biases; some model the baseline cancer risk from all biopsies in the control arm, which include end-of-study biopsies; and others use Surveillance Epidemiology End Results rates to better approximate real-world incidence. Overall, most conclude that a strategy whereby all men over 55 years of age are recommended to take finasteride is not cost-effective [42,43]. However, studies analyzing a strategy targeting only men at higher risk of prostate cancer suggest that finasteride chemoprevention is cost-effective [44-47].

The goal of the REDUCE study was to examine 5-ARIs in men at higher risk in hopes of answering that question. As it turned out, that cohort of men with a prior negative biopsy actually had a risk of prostate cancer equal to that of the PCPT cohort (placebo cancer rate 24.4% in PCPT vs. 25.1% in REDUCE). Only one study has examined the cost-effectiveness of dutasteride based on the REDUCE data and similarly concluded that it is unlikely to be cost-effective unless targeted at a high-risk population [48].

## Future directions

Given the tremendous potential benefits of chemoprevention, there is logic in searching for a different role for 5-ARIs. Indeed, more derivative questions are still being addressed in trials. For example, investigators are studying whether short courses of finasteride improve the discriminating ability of PSA in prostate cancer screening [49] or the cancer yield at repeat biopsy after a prior negative biopsy [50], as well as whether dutasteride prevents cancer in men with high-grade prostate intraepithelial neoplasia, a precursor lesion to prostate cancer [51]. Aside from these trials, more work is needed to identify a subgroup of patients, using either clinical or genetic features, who are at increased risk of prostate cancer and/or are most likely to respond to 5-ARI therapy. Perhaps we can identify a genetic signature that predicts those prone to developing high-grade disease in the dihydrotestosterone-depleted prostate environment, and physicians could avoid using 5-ARIs in these men. Selective treatment in men without this signature would be "safer" in that it only reduces prostate cancer risk without increasing high-grade disease.

It may be that 5-ARIs are more appropriate for secondary prevention, that is, in preventing adverse outcomes in men who already have been diagnosed with cancer. The Reduction by Dutasteride of Clinical Progression Events in Expectant Management (ReDEEM) trial has now concluded. Presented only in abstract form to date, this study illustrated that in men with very low-risk prostate cancer treated with active surveillance, dutasteride reduced the time to pathologic or therapeutic progression by 38.9% (95% confidence interval 12.4 to 57.4; *P *= 0.007) [52].

## Conclusions

With the risk of prostate cancer so high, there is great need for a strategy to reduce the incidence and thus the burden of prostate cancer. Chemoprevention holds such potential in this regard. Yet, the future of 5-ARIs, the most promising chemopreventive agents to date, is uncertain. In the role of wide-scale use to prevent prostate cancer in men of average risk, this is likely the end for 5-ARIs with no further primary prevention trials on the horizon. The widespread acceptance of statins and aspirin for cardiovascular disease prevention proves that patients are willing to take a drug to prevent a disease they may never get. In the case of 5-ARIs, it is likely not the small risk of reversible sexual side effects or the preferential prevention of low-grade disease that are preventing FDA approval and wider adoption. It is the lingering uncertainty surrounding the risk of high-grade disease. No physician or regulatory body is comfortable treating healthy men with a drug that has even the slightest risk of inducing a potentially lethal cancer.

Until more is learned, physicians are unfortunately left in the difficult position of explaining the complicated risks and benefits of 5-ARIs, and while they were never approved for chemopreventive use before, there was always the hope that they would be. Now the hope of their approval is gone, and the scientific community, while still endeavoring to identify a specific subgroup of men who will benefit from 5-ARIs, should begin turning the page toward the next chemoprevention strategy.

## Abbreviations

5-AR: 5-α reductase enzyme; 5-ARI: 5-α-reductase inhibitor; BPH: benign prostatic hyperplasia; DRE: digital rectal examination; FDA: US Food and Drug Administration; NSAID: nonsteroidal anti-inflammatory drug; ODAC: Oncology Drugs Advisory Committee; PCPT: Prostate Cancer Prevention Trial; PSA: prostate-specific antigen; RCT: randomized; controlled trial; REDUCE: REduction by DUtasteride of Prostate Cancer Events; RRR: relative risk reduction.

## Competing interests

RJH has no disclosures. SJF is a paid consultant for GlaxoSmithKline.

## Authors' contributions

Both RJH and SJF contributed equally to the manuscript. Both authors read and approved the final manuscript.

## Pre-publication history

The pre-publication history for this paper can be accessed here:

http://www.biomedcentral.com/1741-7015/9/105/prepub
